# Signaling Cascades Modulate the Speed of Signal Propagation through Space

**DOI:** 10.1371/journal.pone.0004639

**Published:** 2009-02-27

**Authors:** Christopher C. Govern, Arup K. Chakraborty

**Affiliations:** 1 Department of Chemical Engineering, Massachusetts Institute of Technology, Cambridge, Massachusetts, United States of America; 2 Department of Chemistry, Massachusetts Institute of Technology, Cambridge, Massachusetts, United States of America; 3 Department of Biological Engineering, Massachusetts Institute of Technology, Cambridge, Massachusetts, United States of America; Fondazione Telethon, Italy

## Abstract

**Background:**

Cells are not mixed bags of signaling molecules. As a consequence, signals must travel from their origin to distal locations. Much is understood about the purely diffusive propagation of signals through space. Many signals, however, propagate via signaling cascades. Here, we show that, depending on their kinetics, cascades speed up or slow down the propagation of signals through space, relative to pure diffusion.

**Methodology/Principal Findings:**

We modeled simple cascades operating under different limits of Michaelis-Menten kinetics using deterministic reaction-diffusion equations. Cascades operating far from enzyme saturation speed up signal propagation; the second mobile species moves more quickly than the first through space, on average. The enhanced speed is due to more efficient serial activation of a downstream signaling module (by the signaling molecule immediately upstream in the cascade) at points distal from the signaling origin, compared to locations closer to the source. Conversely, cascades operating under saturated kinetics, which exhibit zero-order ultrasensitivity, can slow down signals, ultimately localizing them to regions around the origin.

**Conclusions/Significance:**

Signal speed modulation may be a fundamental function of cascades, affecting the ability of signals to penetrate within a cell, to cross-react with other signals, and to activate distant targets. In particular, enhanced speeds provide a way to increase signal penetration into a cell without needing to flood the cell with large numbers of active signaling molecules; conversely, diminished speeds in zero-order ultrasensitive cascades facilitate strong, but localized, signaling.

## Introduction

Signaling cascades, series of molecules that sequentially activate each other, are ubiquitous in cellular systems 1–4. They have long been thought to amplify input signals as each molecule in the cascade can serially activate multiple molecules of a downstream component of the cascade [5], [6]. However, doubts have been raised about whether cellular conditions actually allow for this [6]. Cascades have also been considered to modulate the duration and timing of signals, filter noise, and otherwise regulate cellular decisions [6]–[8].

The speed of signal propagation through space is also important. For example, how quickly signals propagate though the cell might affect integration of signals from different receptors on the same cell. Insights into the signal amplitude, duration, and timing at points distal from a signal's source cannot be obtained from computational models that treat the system to be homogenous (or well-mixed).

The influence of cascades on the spatial propagation of signals has been considered before [8]–[16]. Much of this work has focused on the long time behavior of spatially inhomogeneous systems or on the kinetics of particular pathways. In the latter case, for example, many studies have focused on the MAPK cascade, a ubiquitous cellular pathway. The MAPK cascade has been shown to enhance signal penetration into the cell, reducing sharp signaling gradients otherwise caused by phosphatase deactivation of the signal as it travels away from the origin [8], [12]. However, according to these studies, simple kinetic considerations do not account for how the cascade enables penetration from the membrane to the nucleus. A more complicated model of the MAPK cascade, involving feedback-induced bistability, has been shown to generate fast-moving signaling waves that might account for long-range propagation [9], [13], [14].

Here, we have examined the mechanistic principles underlying how simple cascades can influence the speed of signal propagation through space regardless of whether the cascade is an intrinsic amplifier or attenuator of signal amplitude.

We find that, depending upon the pertinent kinetic parameters, cascades can either speed up or slow down signal propagation though space in a manner that is largely uncoupled from its impact on features such as amplification of the amplitude. In particular, cascades operating far from saturation can speed signal propagation through the cell. Although phosphatase levels modulating certain kinase cascades have been suggested to be too large for signal penetration into the nucleus, our results may be applicable to kinase cascades over shorter length scales or to other cascaded signaling modules. Additionally, we find that cascades operating under zero-order ultrasensitivity [17], in which the cascaded signal is either completely activated or not active at all, can serve to slow down signal propagation in a cell, even as the signal is amplified overall. By extending to the spatial domain studies that productively used moment analysis in the temporal domain [6], we provide a way to summarize the complex spatiotemporal behaviors of cascades.

## Results and Discussion

### Simple model of a signaling cascade

**Figure 1:**
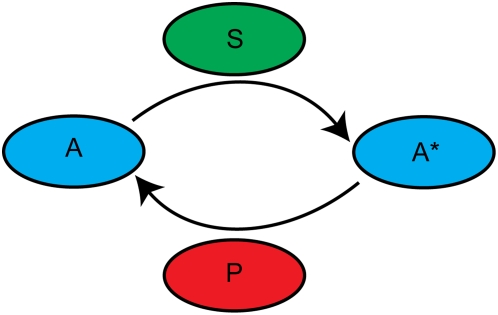
Diagram of a one-level cascade.

We initially model a simple one-level cascade (Figure 1) in which a primary signal, initially localized in space, diffuses away from its origin and activates a secondary, homogenously distributed messenger. Homogenously distributed phosphatases deactivate the signals. In order to reduce the number of competing length scales in the problem, all molecules are assumed to diffuse at identical rates. We neglect many effects that are undoubtedly important, including the effects of scaffolds [6], [18], [19] and feedback regulation [13], [20].

Complications arise in modeling the specific geometries involved in cellular signaling. To keep the discussion general to a variety of length scales and signaling contexts, we model a system of infinite extent in all directions from the initial signal. In other words, we imagine that the distance from the origin of the signal to its ultimate target is large compared to other length scales in the problem. The models have been studied in one dimension.

The primary signal is introduced to the system as a bolus at the origin, as opposed to introduction via a flux, eliminating a time scale in the problem.

Our model differs from more commonly studied models of cascades, in which the primary signal is permanently localized to the origin [8], [10], [12], [15]. Under certain conditions, our model of a one-level cascade is similar to a two-level model in which the primary signal is permanently localized. In particular, the common model collapses to our model if the activation of the first mobile messenger is fast compared to its diffusion time and the reaction time scale. We do not focus on the two-level cascade directly because each level of cascading adds complexity to the problem; our goal is merely to determine whether a secondary mobile messenger travels faster or slower than a primary mobile messenger.

We describe the results of relevant modifications to this simple model throughout the discussion.

### Deterministic formulation of the model

The spatiotemporal evolution of the primary signal, S, and the activated secondary signal, A*, can be described by the following dimensionless (scaled using Table 1) reaction-diffusion equations:
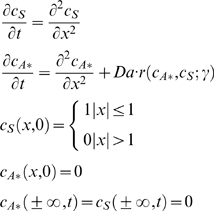
(1)The rate expression *r* incorporates the effect of phosphatases through the parameter γ, as detailed below.

**Table 1:** Scalings in Equation 1.

*Variable*	*Scale*
Distance (*x*)	Characteristic width of the initial primary signal distribution, *L*.
Primary signal concentration (*c_S_*)	Characteristic one-dimensional concentration of the primary signal, *N_S0_/L*.
Secondary signal concentration (c_A*_)	Initial concentration of inactive secondary signal, *c_A0_*.
Time (*t*)	Characteristic diffusion time (*L^2^/D*).
Reaction rate	Characteristic reaction time, dependent on the particular kinetics. See examples in text.

The Damkohler number, *Da*, is the ratio of the diffusion and reaction time scales. Specific forms are given below, as part of the discussion on particular kinetics. If all reactions in the system occur on the same time scale, the Damkohler number compares the time scale over which the primary signal diffuses away from its origin to the time scale over which it begins to activate the secondary signal. In this respect, it measures the significance of the primary signal's localization. For example, if the Damkohler number is small (diffusion is fast compared to reaction), the primary signal delocalizes quickly, before it attempts to react.

In this paper, we study two limits of Michaelis-Menten kinetics for the rate expression r(c_A*_,c_S_; γ). If the enzyme kinetics are far from saturation (the Michealis constants are large relative to the secondary signal concentration), it suffices to consider direct reactions between the secondary signal and its activators and deactivators according to mass action kinetics:
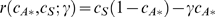
(2)


If the enzyme kinetics are saturated (the Michaelis constants are small), the kinetics of the one level cascade become independent of the secondary signal's concentration:
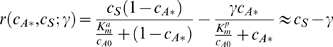
(3)


The expressions in Equations 2 and 3 correspond to Damkohler numbers of 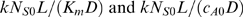, respectively, where *k* and *K_m_* (assuming, for notational simplicity, identical 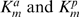) are the constants corresponding to Michaelis-Menten kinetics.

In both limits of the kinetics, the parameter incorporating phosphatase effects, γ, is 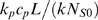, where *k_p_* is the rate constant describing the phosphatase reaction and *c_p_* is the phosphatase concentration. This parameter compares the initial deactivation and activation rates at the signaling origin.

Note that the concentration profile for the primary signal concentration, as described by Equation 1 is just a Gaussian centered at the origin with a variance of *2t*.

To quantify the mean speed of signal propagation, we have analyzed the mean squared displacement of each signal from the origin as a function of time. For the primary signal, S, the mean squared displacement, 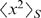, is just *2t*. For the secondary signal, A*, it can be calculated from the concentration profile as:
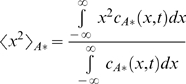
(4)


The variance of the signal's distribution, along with the overall amount of the signal in the system, serves as a summary of its spatiotemporal evolution. The first passage time distribution, also of interest, is less easily discussed deterministically. Also, it is not as decoupled from other functions of the cascade, such as signal amplification: merely amplifying a signal tends to decrease the first passage time, independent of any effect on the signal's propagation speed.

### Cascades operating far from saturated kinetics (large Michaelis constants) speed up signal propagation

**Figure 2:**
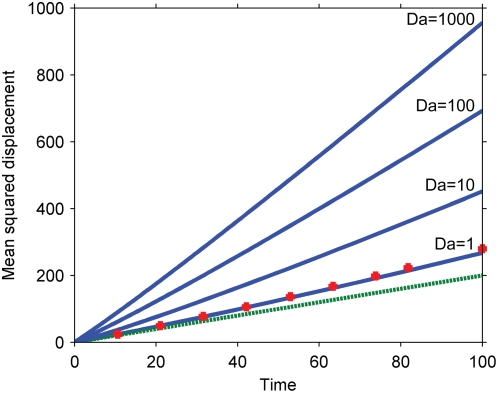
Mean squared displacement of the secondary signal under the kinetics of Equation 2. Dashed line, pure diffusion reference corresponding to the primary signal; solid lines, mean squared displacement of the secondary signal for various Damkohler numbers; squares, approximation for *Da = 1*, corresponding to Equation 5.

Numerical solutions for the mean squared displacement of the secondary signal under the kinetics of Equation 2 are presented in Figure 2 for various values of the Damkohler number, 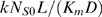, without phosphatases (*γ = 0*). An approximate perturbative solution in the absence of phosphatases, obtained by modeling the initial primary signal as a delta function of unit characteristic length, provides an analytical description for short times and low Damkohler numbers:
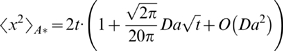
(5)


The numerical and approximate solutions indicate that cascades described by Equation 2 speed up signal propagation; the secondary signal travels faster than the primary. Furthermore, the cascade's effect on the speed of the signal is independent of its effect on the overall amplitude of the signal: the amplitude can be independently controlled by altering the initial, inactive concentration of the secondary signal, c_A0_, which does not affect the signal speed. The enhancement of signal speed is negligible when the Damkohler number is negligible (e.g. for fast diffusion or weak primary signals) and increases as the Damkohler number increases. The seemingly linear increase of the secondary signal's mean squared displacement with time admits the possibility of an effective diffusivity.

Both the increased speed of the secondary signal and its dependence on the Damkohler number can be understood by considering the effects of signal localization on serial triggering. Primary signaling molecules initially localized to the origin must compete with each other there to activate a limited amount of secondary signal, constraining any individual molecule's ability to serially activate many secondary signaling molecules. Primary signaling molecules that diffuse away from the origin, on the other hand, encounter less competition and can more readily serially activate many molecules. Serial triggering is enhanced far from the origin, and the distribution of the secondary signal is shifted to greater distances than the primary signal. In the context of signal speed, the result is that the secondary signal moves faster than its predecessor. For example, if the reaction is instantaneous relative to diffusion (*Da≫1*), the secondary signal becomes fully activated wherever there is at least one molecule of the primary signal – potentially quite far from the origin and certainly further at any given time than the primary signal, on average.

Our results also suggest that the greater the disparity between serial triggering at the origin and far away, the greater the enhancement in the signal's speed. Specifically, the speed increases with the Damkohler number, which measures the importance of a signal's localization. When the Damkohler number is high, the primary signal attempts to react before it diffuses away from the origin, and near the origin, its ability to serially trigger is limited.

There are several implications for these results. Directly, by examining the contributions to the Damkohler number, our results suggest that one effect of strongly stimulating a primary signal (increasing *N_S0_*) is to generate a quickly moving, not just stronger, secondary signal.

**Figure 3:**
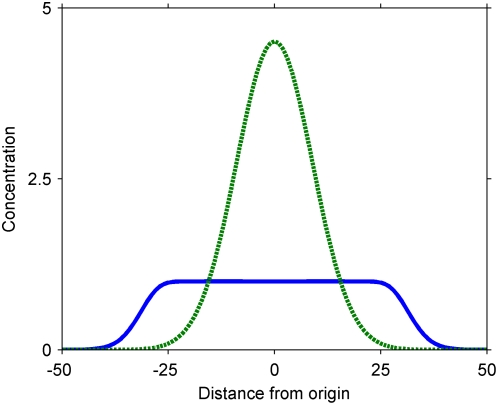
Representative concentration profiles in a cascade that attenuates the overall signal while amplifying the signal far from the origin. Dashed curve, primary signal; solid curve, secondary signal. Parameters (arbitrary units): *Da = 1000*; *c_A0_ = 1*; *N_S0_/L = 100*; *t = 40*. The parameters have been chosen to highlight the limited serial triggering at the origin, where the secondary signal is already, by the figured time, entirely activated.

Our results also clarify previous work indicating that cascades help signals penetrate into a cell [8], [12]. Specifically, we note that one way cascades help signal penetration is by increasing signal speed. This effect is independent from any overall amplification of the primary signal, which would also contribute to increased penetration. In particular, because of the increased speed, a cascade can help a signal penetrate deep into a cell even if it attenuates the overall level of the signal (Figure 3). Cascades provide a way to increase penetration at any given time without flooding the cell with large numbers of active signaling molecules.

Another implication of our results is that cascades do not necessarily cause signaling delays. In homogenous systems, cascades lead to delays, because species buried within the chain take time to become activated [6]. Heterogeneously, however, the secondary signal travels faster than the primary signal, so there may be no delay in its arrival at a target.

We investigated several modifications to our simple model to determine whether the basic conclusions continue to hold in more realistic situations. We find that in all cases cascades described by the kinetics in Equation 2 increase the speed of signal propagation.

**Figure 4:**
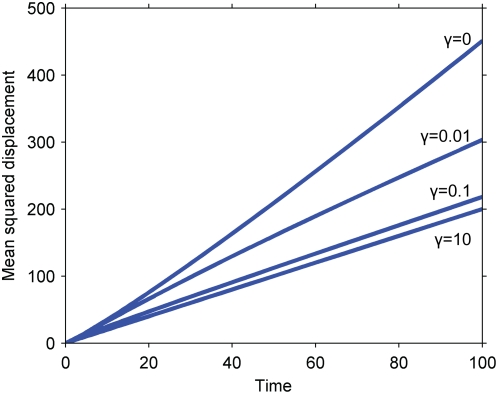
Effect of phosphatases deactivating the secondary signal. The curves indicate the mean squared displacement of the secondary signal for *Da = 10* and various values of the parameter *γ*. The curve for *γ = 10* overlays the purely diffusive curve of the primary signal. Note that *γ* is a parameter that reflects phosphatase activity at the origin only and so understates phosphatase activity in the system as a whole. Similar results pertain to the effect of phosphatases on the primary signal.

For example, we considered the effect of adding phosphatases to the system (Figure 4). These molecules homogenously deactivate the primary and secondary signals. Because continual deactivation at the origin enables serial triggering there, the secondary signal slows down in the presence of phosphatases. Consistent with our previous results, however, the secondary signal still moves faster than the primary signal.

**Figure 5:**
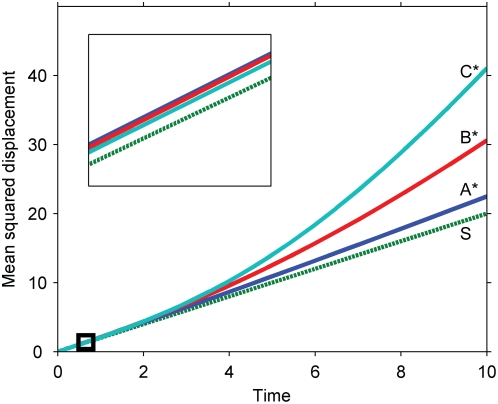
Signal propagation in a multi-level cascade. The cascade ordering is S, A, B, C, with each species activating the species after it in the chain. The parameters have been chosen representatively so that the signal has been amplified at each step by the final time point. (Inset) The cascade at early times, when the signal has not yet been amplified at any level. Note that the species rank, from fastest to slowest, as C*, B*, A*, S at late times (when the signal has been amplified) but as A*, B*, C*, S at early times (when the signal has been attenuated). Parameters: all species are assumed to diffuse at the same rate; the plots correspond to Damkohler numbers of 1 for all levels of the cascade, where the Damkohler number for the i^th^ cascade level is 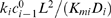.

We also investigated multi-level cascades to determine whether speeds continue to be enhanced as more species are added to a signaling chain. We find, consistent with our previous results, that active signaling molecules at all levels of a cascade travel faster than the primary signal (Figure 5). Furthermore, the same basic features that govern signal speed in a one level cascade seem to govern the speeds at each level in a multi-level cascade. In general, any given step in a multi-level cascade is just a one level cascade in which the primary signal is no longer a simple Gaussian. The language of localization developed above for a Gaussian input, for which the diffusion and reaction time scales determine differences in serial triggering near and far from the origin, broadly translates to multi-level cascades, as suggested by the simulations in Figure 5. If the localization at one level is significant, the next level moves quickly relative to pure diffusion; otherwise, the next level moves almost diffusively. Practically, the consequence for multilevel cascades is that, if signals in the cascade become more and more localized down the chain (e.g. the cascade amplifies signal amplitude, reducing reaction times), the signal travels more and more quickly; if the signals become less localized (e.g. the cascade attenuates signal amplitude), the speeds tend toward pure diffusion.

**Figure 6:**
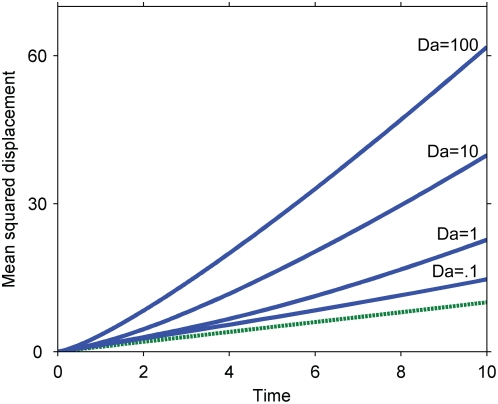
Effect of generating the primary signal at a constant rate at the origin. The results are parameterized by a Damkohler number equal to 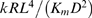, where *R* is the rate of generation at the origin. Dashed line, primary signal; solid lines, secondary signal for different values of the Damkohler number.

We also interrogated our assumption that the primary signal enters the system instantaneously as a bolus. In many contexts, the primary mobile signal in a cascade is activated over time by a permanently localized predecessor (e.g. one bound to the membrane). To investigate the consequence of this, we considered a model in which the primary mobile signal is generated at the origin at some constant rate (Figure 6). Consistent with our previous results, the secondary signal travels faster than the primary signal. In addition, we investigated a more detailed model in which the primary mobile signal, initially inactive and homogenously distributed, is activated by a signal on the membrane that decays exponentially over time (Supplementary Figure S1a). Again, the secondary mobile signal travels faster than its predecessor. Furthermore, if the membrane-bound signal decays rapidly, quickly activating the primary signal, the results coincide with those of our simple model (Supplementary Figure S1b).

**Figure 7:**
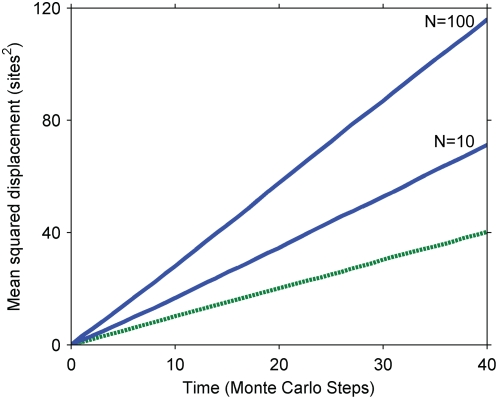
Mean squared displacement of the primary and secondary signals, simulated stochastically. The results are from Monte Carlo simulations on a one-dimensional lattice. At each time step, all molecules hop to adjacent sites and react, if possible, as described in Text S1. Dashed line, purely diffusive reference corresponding to the primary signal; solid lines, secondary signal for different numbers of primary signaling molecules initially at the origin (*N*). The results are independent of the number of secondary signaling molecules in the system.

Finally, because the mean-squared-displacement metric is sensitive to the tails of the signals' distributions, we conducted Monte Carlo simulations of our original model with finite, integer particle numbers (Figure 7). As in the deterministic simulations, the secondary messenger travels faster than its predecessor in the cascade. The exact scaling with the Damkohler number was not recovered (not shown), possibly because stochastic effects alter the scaling with the number of particles in the system.

In the simple system we investigated, as well as in all the modifications, the cascade serves to speed up the propagation of a signal from its origin. In certain parameter regimes – fast diffusion, slow reaction, strong phosphatases, or weak signals – the difference can be negligible. The kinetics, however, admit the phenomena. In biological systems, in which crowded environments slow down diffusion relative to reaction and phosphatase recruitment is often delayed, the effects we have described are likely to be relevant.

### Cascades operating under zero-order ultrasensitivity lead to signal localization

Cascades operating under the kinetics of Equation 3 exhibit behavior known as zero-order ultrasensitivity [17]: in homogenous systems, the secondary signal is either completely activated or left inactive depending on whether the primary signal exceeds the threshold, *γ*.

**Figure 8:**
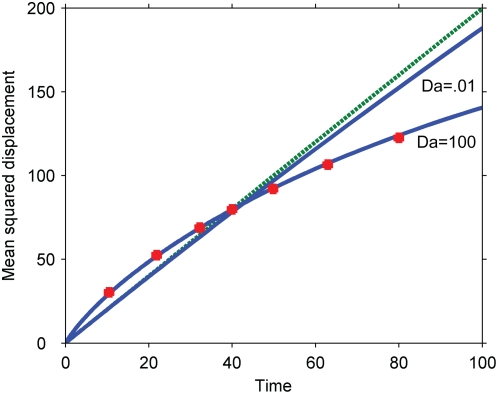
Mean squared displacement of the primary and secondary signals under the kinetics of Equation 3 (zero-order ultrasensitivity). Dashed line, purely diffusive reference corresponding to the primary signal; solid lines, simulation results corresponding to γ = .01 and slow (*Da = 100*) and fast (*Da = .01*) diffusion; squares, theoretical prediction for slow diffusion corresponding to Equation 6. Simulations conducted with *K_m_/c_A0_ = .01* (Equation 3).

Numerical solutions for the propagation speed of the secondary signal under these kinetics are presented in Figure 8 for various values of the Damkohler number. An approximate solution for the mean squared displacement at large Damkohler numbers is:
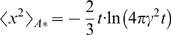
(6)


This approximation is obtained by assuming that the diffusion time is much slower than the reaction time, so that the secondary signal immediately responds to changes in the primary signal's concentration. In this limiting case, a sharp boundary exists between the complete activation of the secondary signal near the origin, where the primary signal exceeds the threshold, and its complete inactivity further away. The secondary signal's mean squared displacement can then be estimated by tracking this threshold concentration in the Gaussian distribution describing the primary signal. Note that the Damkohler number does not appear in Equation 6 as in this approximation it has been assumed to be infinite for the limiting case.

As indicated by the numerical simulations and by the approximate solution, cascades operating under zero-order ultrasensitivity can both speed up a signal (at early times) and slow it down (at later times). Eventually, the primary signal is nowhere above the threshold and the secondary signal, after contracting, entirely disappears. Similar to our results in the previous section, when the Damkohler number is small, diffusion dominates and the corrections to pure diffusive motion disappear.

Because the overall concentration of the inactive secondary signal (*c_A0_*) appears in the Damkohler number, the signal propagation speed is no longer completely decoupled from the amplification effects of the cascade. In particular, any attempt to drastically amplify the primary signal will promote purely diffusive motion of the secondary signal, because the system's tendency to remove sharp gradients washes out all other effects. The speed and signal amplitude are still independent in the important sense that the signal can be slowed down independently of whether it is also amplified or attenuated, depending on the parameters.

These results have several implications. Like the cascades studied in the previous section, zero-order ultrasensitive cascades can be used to speed up signal propagation. A unique feature of these cascades, however, is that they can also slow down signal propagation, eventually confining the secondary signal to a region around the signaling origin and preventing it from reaching any distant targets or interacting with distant signals. The confinement is accomplished purely by the kinetics of the reactions. The region to which the signal is confined, corresponding to the maximum possible mean squared displacement of the secondary signal, can be approximated as:
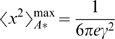
(7)


As expected, phosphatases shrink the region over which the signal can propagate.

Because they can slow down signal propagation in space without necessarily attenuating a signal, cascades under the kinetics of Equation 3 provide a way for generating strong signals without promoting cross-reactivity with distant signals or interference with distant targets. Other simple mechanisms do not simultaneously localize and amplify the signal. For example, a strongly stimulated, uncascaded signal would necessarily lead to increased penetration and interference within the cell; a signal localized solely by strong phosphatase activity would be commensurately weakened.

**Figure 9:**
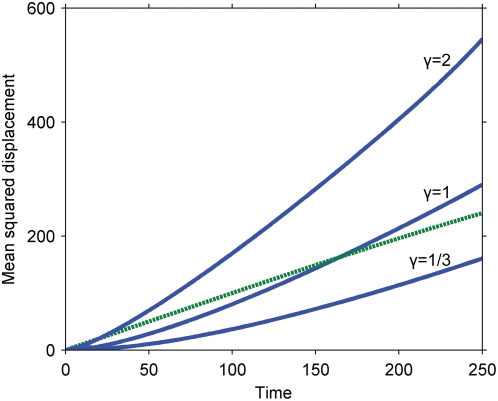
Effect of generating the primary signal continuously at the origin. The results are parameterized by *γ = Dk_p_c_p_/(kRL^2^)*, where *R* is the rate of formation of the primary signal at the origin (see Text S1). The parameters have been chosen so that reactions are fast compared to diffusion (the Damkohler number is approximately infinite). Dashed line, primary signal; solid lines, secondary signal for different values of *γ*.

We investigated our simplification that the primary signal enters the system instantaneously as a bolus. If, instead, the primary signal is generated at a constant rate at the origin, the secondary signal moves more slowly than the primary signal at early times and moves more quickly at later times, a temporal order that is opposite that of the original result (Figure 9). If, additionally, phosphatases are added to the system to deactivate the primary signal, the mean squared displacements eventually plateau as a steady state is reached between generation and destruction of the primary signal (Supplementary Figure S2). At long times, the secondary signal will either be more or less localized than the primary signal depending on whether the steady state is reached while the secondary signal moves slower or faster than the primary signal. If the phosphatases are strong and the system quickly reaches steady state, the secondary signal remains more localized than the primary signal; otherwise, it remains less localized. Given that the novel feature of cascades operating under zero-order ultrasensitivity is that they can slow down signal propagation, the relevance of our results in these modified models depends on whether the early period of slowing down is long compared to other signaling processes, such as phosphatase deactivation of the primary signal.

**Figure 10:**
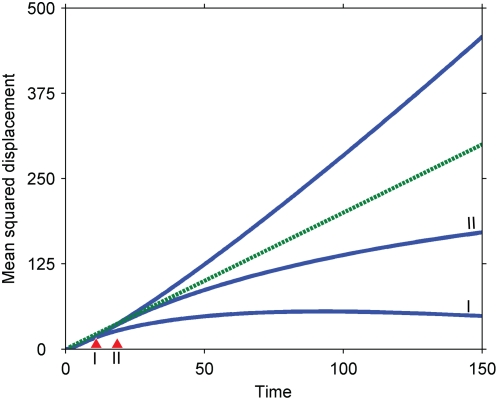
Effect of cutting off generation of the primary signal. Three cases are investigated: the primary signal is not cut off; the primary signal is cut off after a nondimensional time of 5 (I); the primary signal is shut off after a nondimensional time of 10 (II). Dashed line, purely diffusive reference; solid lines, secondary signals corresponding to the three cases. Parameters: *k = 1*; *D = 1*; *R = 10* (rate of generation at origin); *L = 1*; *k_p_c_p_ = 1*. See Text S1 for more details.

Importantly, once the generation of the primary signal is shut off, the system behaves analogously to our simple model: the secondary signal moves more slowly than the primary signal, contracting as the primary signal dilutes (Figure 10). Thus, the results obtained for our simple model appear to apply to more detailed models on time scales longer than the generation of the primary signal.

We hope that our study adds to the framework for thinking about the role of cascades in signal transduction, especially how cascades influence signal propagation in space.

## Supporting Information

Text S1 Equations supplemental to the main text.
(0.05 MB DOC)

Figure S1 Effect of primary signal activation by a decaying, immobile signal. Simulations correspond to Equation S3. (a) Slow decay (τ_decay_ = 10). Dashed line, primary signal (a representative curve is shown for clarity; the three cases are within 10% of this curve); solid lines, secondary signal. The Damkohler numbers of the first and second steps were chosen to be identical for the simulations. (b) Fast decay (τ_decay_ = .001). Parameters chosen so that the primary signal is generated in an initial burst (Da_1_ = 1000; Da_2_ = 1). Dashed line, primary signal; solid line, secondary signal; squares, simulations corresponding to the original model (Equation 1 in the main text) with the initial bolus of signal (N_S0_) set to the amount of primary signal eventually generated in the case of fast decay.
(0.49 MB TIF)

Figure S2 The effect of phosphatases that deactivate the primary signal. Lines with squares, Da_p_ = 0.01; lines with circles, Da_p_ = 0.005; open symbols, primary signal; closed symbols, secondary signals. Other parameters: Da = 1.5; γ = 0.67.
(0.37 MB TIF)
