# Supplementary material for: Signaling Cascades Modulate the Speed of Signal Propagation through Space
Source: PLoS One. 2009 Feb 27;4(2):e4639. doi: 10.1371/journal.pone.0004639 (PMC2645680; doi:10.1371/journal.pone.0004639)
Supplement: Text S1 — Equations supplemental to the main text. (0.05 MB DOC) [file pone.0004639.s001.doc]

**SUPPLEMENT**

**Description of modifications to the simple model**

Several modifications to our basic model are discussed in the text. These are explicitly enumerated below in more detail.

1. The primary signal is constantly generated at the origin.

$\frac{\partial c_{S}}{\partial t}=\frac{\partial^{2}c_{S}}{\partial x^{2}}+\delta(x)$ (S1)

$$\frac{\partial c_{A*}}{\partial t}=\frac{\partial^{2}c_{A*}}{\partial x^{2}}+Da\cdot r\left(c_{A*},c_{S};\gamma\right)$$

$$c_{S}(x,0)=c_{A*}(x,0)=0$$

$$c_{A*}(\pm\infty,t)=c_{S}(\pm\infty,t)=0$$

The Damkohler number corresponds to those in the main text, except that c_s_ is scaled by$RL^{2}/D$, where *R* is the rate of generation at the origin. Specifically, for kinetics far from saturation, *Da=*$kRL^{4}/\left(K_{m}D^{2}\right)$; for zero-order ultrasensitive kinetics, it is $kRL^{4}/(c_{A0}D^{2})$. The parameter *γ* is *Dk_p_c_p_/(kRL^2^)*.

1. The primary signal is constantly generated at the origin; phosphatases homogenously deactivate the primary and secondary signals.

$\frac{\partial c_{S}}{\partial t}=\frac{\partial^{2}c_{S}}{\partial x^{2}}+\delta(x)-Da_{p}c_{s}$ (S2)

$$\frac{\partial c_{A*}}{\partial t}=\frac{\partial^{2}c_{A*}}{\partial x^{2}}+Da\cdot r\left(c_{A*},c_{S};\gamma\right)$$

$$c_{S}(x,0)=c_{A*}(x,0)=0$$

$$c_{A*}(\pm\infty,t)=c_{S}(\pm\infty,t)=0$$

$$Da_{p}=\frac{k_{p}c_{p}L^{2}}{D}$$

The Damkohler number *Da* and the parameter *γ* are as described in the first scenario.

1. The primary mobile signal is generated at the origin by an exponentially decaying signal (I) permanently localized to the origin.

$\frac{\partial c_{S}}{\partial t}=\frac{\partial^{2}c_{S}}{\partial x^{2}}+Da_{1}e^{-\frac{t}{\tau_{decay}}}\cdot(1-c_{s})\cdot\delta(x)$ (S3)

$$\frac{\partial c_{A*}}{\partial t}=\frac{\partial^{2}c_{A*}}{\partial x^{2}}+Da_{2}\cdot r\left(c_{A*},c_{S};\gamma\right)$$

$$c_{S}(x,0)=c_{A*}(x,0)=0$$

$$c_{A*}(\pm\infty,t)=c_{S}(\pm\infty,t)=0$$

$$Da_{1}=\frac{k_{s}N_{I}L}{K_{m}^{s}D}$$

$$Da_{2}=\frac{k_{a}c_{S0}L^{2}}{K_{m}^{a}D}$$

In these equations, *N_I_* is the initial amount of the signal I in the region of size *L*; *c_S0_* is the initial amount of inactive S in the system; $\tau_{decay}$is the ratio of the decay time of I to the diffusion time.

For kinetics far from saturation (Equation 2 in the main text), we also conducted stochastic simulations with finite particle numbers. These were simple Monte Carlo simulations on a one dimensional grid with no excluded volume. The grid was initialized with *N_S0_* molecules of the primary signal at the origin and the requisite number of inactive secondary signaling molecules randomly distributed throughout the system to achieve a number concentration of *c_A0_*. At each time step, every molecule was given a chance, in random order, to hop to an adjacent site and, separately, to react, if possible. (This corresponds to a case of commensurate reaction and diffusion propensities.)
