# Supplementary figures and images for: Signaling Cascades Modulate the Speed of Signal Propagation through Space
Source: PLoS One. 2009 Feb 27;4(2):e4639. doi: 10.1371/journal.pone.0004639 (PMC2645680; doi:10.1371/journal.pone.0004639)

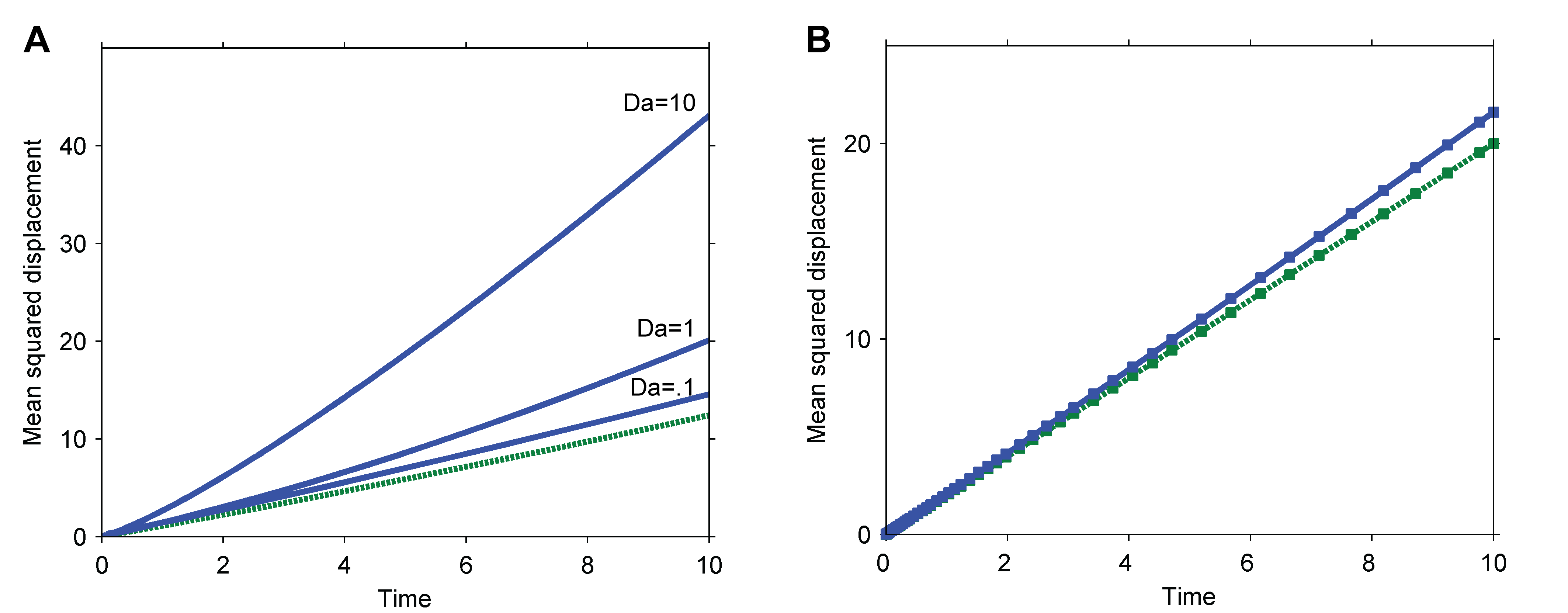

Supplement: Figure S1 — Effect of primary signal activation by a decaying, immobile signal. Simulations correspond to Equation S3. (a) Slow decay (τdecay = 10). Dashed line, primary signal (a representative curve is shown for clarity; the three cases are within 10% of this curve); solid lines, secondary signal. The Damkohler numbers of the first and second steps were chosen to be identical for the simulations. (b) Fast decay (τdecay = .001). Parameters chosen so that the primary signal is generated in an initial burst (Da1 = 1000; Da2 = 1). Dashed line, primary signal; solid line, secondary signal; squares, simulations corresponding to the original model (Equation 1 in the main text) with the initial bolus of signal (NS0) set to the amount of primary signal eventually generated in the case of fast decay. (0.49 MB TIF) [file pone.0004639.s002.tif]

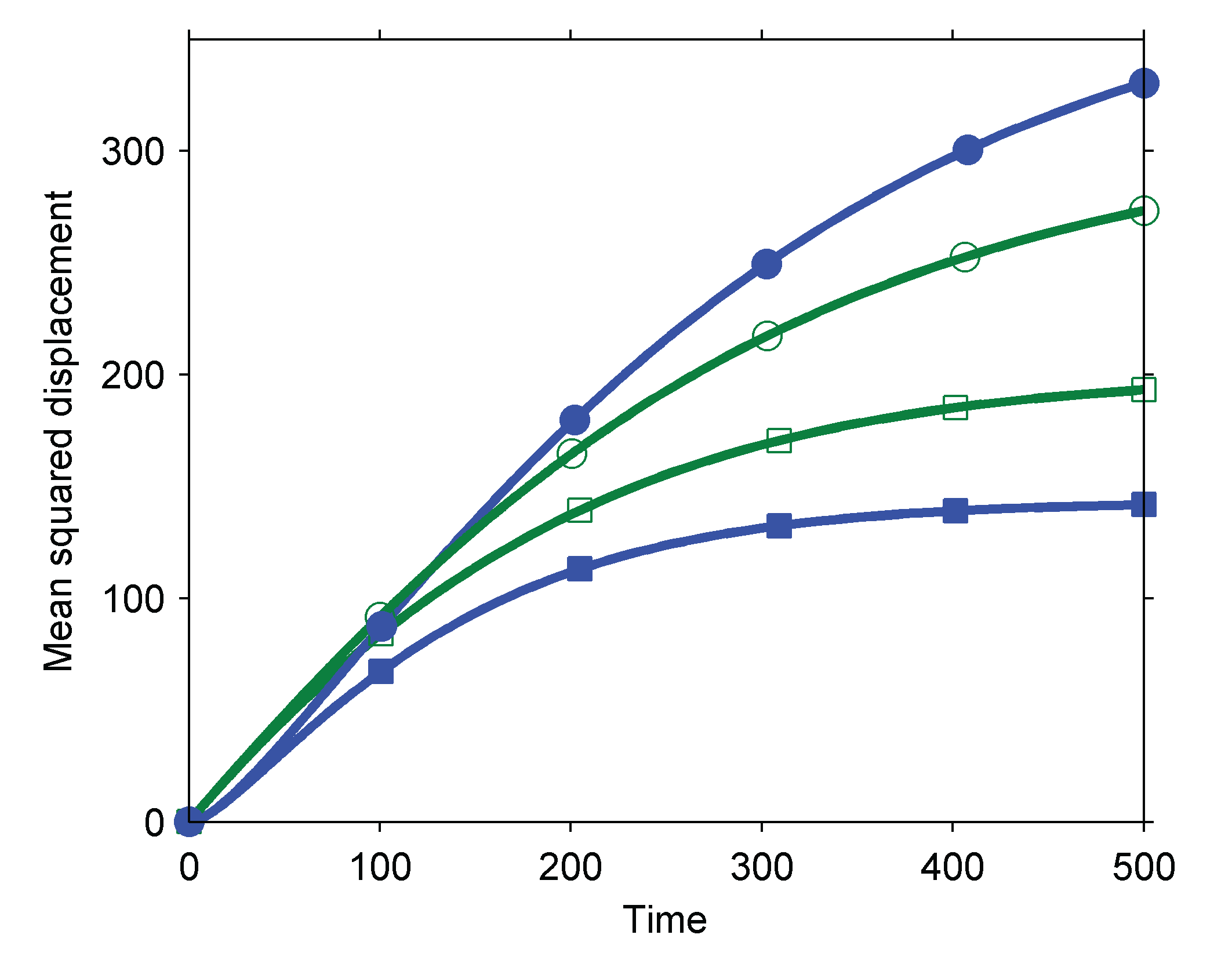

Supplement: Figure S2 — The effect of phosphatases that deactivate the primary signal. Lines with squares, Dap = 0.01; lines with circles, Dap = 0.005; open symbols, primary signal; closed symbols, secondary signals. Other parameters: Da = 1.5; γ = 0.67. (0.37 MB TIF) [file pone.0004639.s003.tif]
